# Digoxin enhances radiation response in radioresistant A549 cells by reducing protein phosphatase 2A

**DOI:** 10.1042/BSR20171257

**Published:** 2017-11-29

**Authors:** Ji Young Lee, Mi-Sook Kim, Mi So Lee, Jae Eun Ju, Namhyun Chung, Youn Kyoung Jeong

**Affiliations:** 1 Radiation Non-clinical Center, Korea Institute of Radiological and Medical Sciences, Seoul 01812, Republic of Korea; 2 Department of Radiation Oncology, Korea Institute of Radiological and Medical Sciences, Seoul 01812, Republic of Korea; 3 Department of Biosystems Engineering, College of Life Sciences and Biotechnology, Korea University, Seoul 02841, Republic of Korea

**Keywords:** Cardiac glycosides, PP2A inhibitor, radioresistance, radiosensitizer

## Abstract

Protein phosphatase 2A (PP2A) is a ubiquitous multifunctional enzyme usually known as a tumor suppressor. Recent studies have reported that although inhibition of PP2A leads to acceleration of cell growth, it also induces damaged cells to pass through the cell cycle and renders them sensitive to radiotherapy. Here, we investigated the radiosensitizing effects of digoxin as a PP2A inhibitor in two non-small-cell lung cancer (NSCLC) cell types (H460 and A549) with differential sensitivity to radiation. Digoxin inhibited the proliferation of H460 and A549 cells in a dose-dependent fashion and was especially effective on radioresistant A549 cells. Interestingly, the radiosensitizing effect of digoxin was only present in the radioresistant A549 cells and xenografts. The combination of digoxin and ionizing radiation (IR) significantly reduced clonogenic survival and xenograft tumor growth (*P*<0.001), compared with IR alone. Digoxin suppressed PP2A protein expression and prevented IR-induced PP2A expression in A549 cells. Digoxin treatment combined with IR allowed the damaged cell to progress through the cell cycle via suppression of cell cycle-related proteins (p53, cyclin D1, cyclin B1, CDK4, and p-cdc2). Moreover, digoxin enhanced IR-induced DNA damage through reduction in levels of repair proteins and elevation of p-ATM foci formation up to 24 h (*P*<0.001). In conclusion, digoxin has a novel function as a PP2A inhibitor, and combined with IR produces a synergistic effect on radiosensitizing cells, thereby indicating a potentially promising therapeutic approach to radioresistant lung cancer treatment.

## Introduction

Lung cancer is the leading cause of cancer mortality in both males and females worldwide [1]. In early stages, treatment of non-small-cell lung cancer (NSCLC) is mostly surgery. While, in inoperable late-stage NSCLC, radiotherapy plays a crucial role in the management of lung cancer [2]. However, a major contributor to radiotherapy failure is radioresistance, which is associated with specific cellular mechanisms including prosurvival signaling pathways, cell cycle checkpoint regulation, DNA damage repair pathway, epithelial–mesenchymal transition, and inflammation [3–5]. Thus, modulation of these mechanisms provides a promising alternative approach for enhancing radiosensitivity.

Protein phosphatase 2A (PP2A), a ubiquitous and multifunctional serine-threonine phosphatase, plays a critical role in cellular processes such as cell cycle progression, DNA replication, signal transduction, apoptosis, and invasiveness [6,7]. Depending on the physiological needs of the cell, PP2A acts as a tumor suppressor or tumor promoter by inducing the conversion of its target proteins from a transiently phosphorylated state into a dephosphorylated state. Focussing on its role as a ‘tumor suppressor’, inhibition of PP2A induces tumorigenesis by phosphorylation of components of pathways involved in cancer cell growth, such as extracellular signal related kinase (ERK), p38, Akt, and NF-κB [8,9]. However, emerging evidence suggests that inhibition of PP2A induces anticancer effects by sustained phosphorylation of p53, γH2AX, and ATM leading to apoptosis, cell cycle deregulation, and inhibition of DNA repair [10–12]. Moreover, PP2A inhibitors sensitize cells to radiotherapy by enhancing ionizing radiation (IR) induced mitotic catastrophe, reducing DNA damage repair, and diminishing the DNA damage response activity of p53 [13]. Therefore, the use of PP2A inhibition to overcome radioresistance would prove to be a good approach to enhance radiation response in NSCLC.

Recently, Kim et al. [14] found that STK11 mutant NSCLC cells were more sensitive to cardiac glycosides, especially digoxin. Cardiac glycosides are inhibitors of Na^+^/K^+^ ATPase, and are used to enhance cardiac contractility in patients with congestive heart failure and cardiac arrhythmias. According to previous pharmacokinetics/pharmacodynamic studies of cardiac glycosides, digoxin, which is one of cardiac glycosides, is primarily absorbed in small intestine and is eliminated by kidneys [15]. In addition, the pharmacodynamic effects of digoxin are correlated with the uptake of digoxin in the heart after a single dose and with the steady-state serum digoxin concentration during maintenance therapy [16]. Over the past few years, cardiac glycosides have been shown to have applications in cancer treatment [17,18]. In clinical research, patients on *Digitalis* treatment showed better response to anticancer therapy and lower death rates than those who were not on *Digitalis* treatment [19]. Other studies also revealed that cardiac glycosides reduce proliferation and enhance apoptosis in various cancer cells at concentrations that were non-toxic to normal cells [20–22]. Indeed, some cardiac glycosides such as ouabain, oleandrin, and Huachansu enhanced radiosensitivity through inhibition of DNA repair and enhancing IR-induced apoptosis in NSCLC cells [23–25]. In addition, digoxin showed anticancer effects through suppression of Src activity [26] and inhibition of HIF-1α synthesis [27] in NSCLC. However, the radiosensitizing effects of digoxin have not yet been understood fully. In the present study, we investigated whether digoxin would enhance the radiosensitizing effect in NSCLC with particular emphasis on the role of PP2A in cancer.

## Materials and methods

### Drug

Digoxin was obtained from Sigma–Aldrich Chemical Corp. (St. Louis, MO, U.S.A.). Digoxin was dissolved in methanol to a concentration of 4 mM and stored at −20°C.

### Cell cultures

Human NSCLC cell lines H460 and A549 were obtained from the Korean Cell Line Bank (Seoul, South Korea). H460 cells were cultured in Roswell Park Memorial Institute 1640 (RPMI-1640) medium (Welgene, Seoul, South Korea) and A549 cells were cultured in Dulbecco’s modified Eagle’s medium (DMEM) (Welgene, Seoul, South Korea), supplemented with 10% FBS, 100 units/ml penicillin, and 100 μg/ml streptomycin. All cells were cultured at 37°C in a humidified incubator under an atmosphere of 5% CO_2_.

### Irradiation

Cells were irradiated with a ^137^Cs γ-ray source (Atomic Energy of Canada, Ltd., Chalk River, Ontario, Canada) at a dose rate of 2.67 Gy/min. Xenografted mice were irradiated using a ^60^Co γ-ray source (Theratron 780, Atomic Energy of Canada, Chalk River, Ontario, Canada) with a 0.5 cm diameter bolus of tissue equivalent material to allow for dose buildup.

### Water-soluble tetrazolium-1 assays

The cells were seeded in a 96-well plate at a density of 1 × 10^3^ cells per well. Digoxin in varying concentrations (0–120 nM) was added to each well, and the cells were incubated for 48 h, followed by the application of the water-soluble tetrazolium (WST)-1 cytotoxicity assay reagent (EZ-Cytox; DoGen, Seoul, South Korea) according to the manufacturer’s recommendations.

### Colony forming assay

Cells were seeded into 60-mm culture plates and allowed to attach overnight before treatment with 40 nM of digoxin for 24 h before IR, and then further incubated for 24 h. Twelve days after seeding, colonies were fixed with 100% methanol and stained with 0.4% Crystal Violet, and the number of colonies with at least 50 cells was counted.

### p-ATM immunofluorescence assay

Immunofluorescence staining was performed to determine the nuclear distribution of p-ATM foci in H460 and A549 cells using image analysis. Cells were grown on chambered slides 1 day prior to irradiation or digoxin treatments. After digoxin (40 nM) exposure for 24 h, cells were irradiated and incubated for 1 or 24 h before harvest. Cells were fixed with 4% paraformaldehyde, washed with PBS, permeabilized with 0.6% Triton X-100 in PBS, blocked with 4% FBS in PBS, and incubated in blocking buffer containing primary antibody against p-ATM (Santa Cruz Biotechnology, San Diego, CA, U.S.A.) and then incubated with FITC-labeled goat anti-mouse IgG (Invitrogen, Carlsbad, CA). Nuclei were counterstained with DAPI (Sigma, St. Louis, MO). Coverslips were mounted with fluorescence mounting medium. The slides were examined using a fluorescence microscope with digital imaging system (Olympus, Tokyo, Japan) and images were captured with a charge-coupled device camera. For quantitative analysis, foci-positive cells were counted in at least 50 cells from randomly captured images.

### Western blot analysis

Whole cells and homogenized tissue lysates were prepared in cold radioimmunoprecipitation assay (RIPA) buffer supplemented with phosphatase and protease inhibitors. Protein quantity was determined by Bio–Rad Protein Assay. Proteins were separated using SDS/PAGE and transferred to nitrocellulose membranes. The membranes were blocked with 5% (v/v) skim milk in PBS with 0.1% Tween 20, incubated with the indicated antibodies (1:1000) and secondary antibodies (1:1000), and then subsequently developed using ECL Western blotting substrate (Cyanangen Srl, Bologna, Italy) using the ImageQuant LAS-4000 mini (GE, Fairfield, CT, U.S.A.). The signal intensity of the bands was measured with the Multigauge V3.0 software (Fujifilm Life Science, Tokyo, Japan).

### Animal experiments

Athymic Balb/c nude mice (4-week-old males) were purchased from Nara Biothech Co. (Seoul, Korea) and maintained in a laminar airflow cabinet under specific pathogen-free conditions. H460 and A549 xenograft mouse models were established by subcutaneous injection of 3 × 10^6^ H460 or A549 cells into the right thigh. When the tumor attained a volume of approximately 100 mm^3^, the mice were randomly divided into four groups (*n*=6 or 10) including control, digoxin, IR, and combination of digoxin and IR. Digoxin and vehicle were orally administered every 2 days at a concentration of 3 mg/kg.

When the tumor volume of control group attained 200–250 mm^3^, the IR-treated groups (IR alone and digoxin plus IR) were treated with a single 5-Gy fraction of local-regional irradiation using a ^60^Co irradiator. The tumor volume (V) was calculated using the standard formula: V (mm^3^) = π/6 × (smaller diameter)^2^ × (larger diameter). Mice were killed by carbon dioxide (CO_2_) inhalation when the average tumor volume of the control group was 1000 mm^3^.

### Statistical analysis

Statistical analysis was performed using independent *t* test and one-way ANOVA followed by Tukey’s honest significant difference (HSD) test through the Statistical Package for the Social Sciences (SPSS) software (version 23.0, Chicago, IL, U.S.A.).

### Ethics statement

All animal study protocols and studies were approved by the Institutional Animal Care and Use Committee (IACUC) of the Korean Institute of Radiological and Medical Sciences (KIRAMS 2016-53).

## Results

### Digoxin induces more cell death in radioresistant A549 than radiosensitive H460 cells

**Figure 1:**
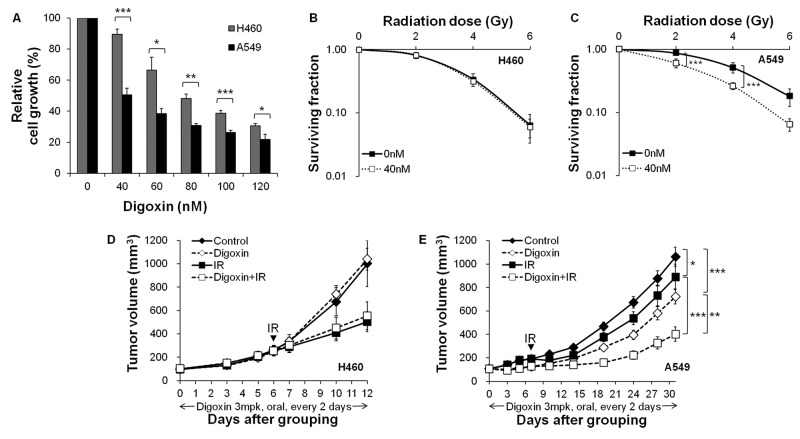
Digoxin inhibits H460 and A549 cell growth and has a radiosensitizing effect in radioresistant A549 cells (**A**) Cell viability determined by WST-1 assay. Cells were treated with vehicle and digoxin in indicated concentrations for 48 h. Each column represents the mean ± S.D. Radiosensitizing effect examined by colony forming assay in H460 (**B**) and A549 cells (**C**). Cells were pretreated with 40 nM digoxin, and then irradiated with different doses of γ radiations. Data shown are mean ± S.D. from at least three independent experiments. Tumor volumes from nude mice bearing xenografts of (**D**) H460 or (**E**) A549 cells. Mice were orally administered with digoxin 3 mg/kg per every 2 days. The control group received vehicle only. Tumor volumes were calculated as described in the ‘Materials and methods’ section. Values represent the mean ± S.E.M; *P<0.05, **P<0.01, ***P<0.001.

To evaluate the effect of digoxin on the cell growth, H460 and A549 cancer cells were exposed to various concentrations of digoxin for 48 h. Digoxin decreased cell growth by 89.5 ± 3.4, 66.4 ± 0.2, 48.2 ± 2.9, 38.7 ± 1.8, and 30.5 ± 1.5% in H460 cells (*P*<0.05) and 50.6 ± 4.3, 38.4 ± 3.4, 30.7 ± 1.2, 26.4 ± 1.4, and 21.8 ± 3.3% in A549 cells (*P*<0.01) at the concentrations of 40, 60, 80, 100, and 120 nM, respectively (Figure 1A). Digoxin suppressed the proliferation of both cells in a dose-dependent manner, and A549 cells were more sensitive to cell death by digoxin than H460 cells were (*P*<0.05).

### Digoxin elevates radiosensitivity only in radioresistant A549 cells and xenograft tumors

To examine the effect of digoxin on the cellular radiation response, we treated with 40 nM of digoxin for 24 h followed by IR, and then removed the drug at 24 h after IR in H460 and A549 cells. The surviving fractions following exposure to a 2 Gy IR were determined to be 0.81 and 0.82 for H460 cells (Figure 1B) and 0.87 and 0.60 for A549 cells (*P*<0.001) (Figure 1C), following treatment with IR alone and combined with digoxin, respectively. Combination of digoxin plus IR did not enhance the clonogenic cell death compared with IR alone in H460 cells, but it raised clonogenic cell death in A549 cells. These results indicated that IR treatment combined with digoxin elevates radiosensitivity in A549 cells, but not in H460 cells.

To validate the radiosensitizing effect of digoxin *in vivo*, H460 and A549 xenografts in athymic nude mice were established. The time required for the volume of control tumors to reach 1000 mm^3^ was 12 days for H460 and 31 days for A549 xenografts, and the growth rate of A549 was markedly slower than H460 xenografts (Figure 1D,E). Single treatment with 5 Gy of IR inhibited tumor growth in both xenograft tumors and significantly suppressed tumor growth in radiosensitive H460 tumors (50.5 ± 8.5%; compared with control) while showing a lower level of suppression in radioresistant A549 tumors (16.3 ± 10.1%, *P*<0.05; compared with control). Treatment with digoxin induced tumor growth inhibition in A549 tumors (31.9 ± 6.1%, *P*<0.001; compared with control), but not in H460 xenograft tumors. Digoxin treatment combined with IR significantly reduced tumor growth in A549 xenograft tumors (54.8 ± 5.7%, *P*<0.001; compared with IR alone), whereas digoxin did not enhance the tumor suppression by IR in H460 xenograft tumors. Oral administration of digoxin for 30 days in normal mice did not cause significant toxicity to mice treated with digoxin (3 mg/kg) as assayed by mouse body weight in comparison with the vehicle-treated control group (results not shown). Therefore, we speculated that digoxin enhances radiosensitivity in radioresistant A549, but not in radiosensitive H460 cells and xenograft tumors.

### Digoxin enhances radiation response via reduction in PP2A expression

**Figure 2:**
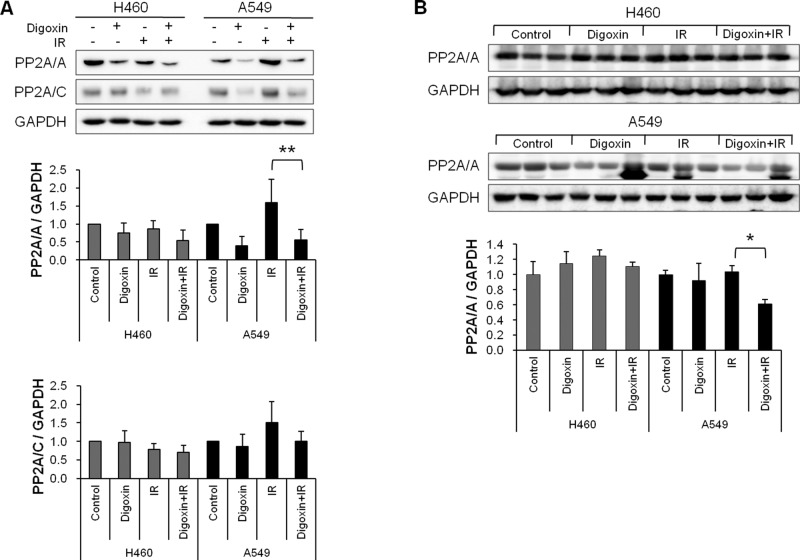
Digoxin reduces PP2A expression in H460 and A549 cells (**A**) Western blot showing protein expression levels of PP2A/A and PP2A/C in cells pretreated with 40 nM digoxin for 24 h, and then irradiated with 6 Gy IR. Bars represent ± S.D.; *P<0.05, **P<0.01. (**B**) Western blot analysis of PP2A protein levels from H460 and A549 xenograft tumor tissues. Tumors were isolated and lysed for Western blotting when the tumor volume of control group reached 1000 mm^3^.

To assess the role of digoxin as a PP2A inhibitor, we examined the expression of PP2A/A and PP2A/C by Western blotting. As shown in Figure 2, the basal protein level of PP2A/A *in vitro* and *in vivo* was 1.6-times higher in H460 than A549 (*P*<0.05). Treatment with digoxin reduced PP2A/A expression in both H460 and A549 cells (Figure 2A). IR raised expression of PP2A/A and PP2A/C only in A549 cells. Digoxin attenuated IR-induced PP2A/A (*P*<0.01) and PP2A/C expression in A549 cells, but not in H460 cells. Additionally, digoxin significantly reduced the IR-induced PP2A protein expression in A549 xenograft tumors but not in H460 xenograft tumors (*P*<0.05) (Figure 2B). These data show that digoxin acts as a PP2A inhibitor and enhances the radiosensitivity of A549 cells and xenografts by reduction in IR-induced PP2A protein levels.

### Digoxin suppresses the expression of cell cycle related proteins in A549 cells

**Figure 3:**
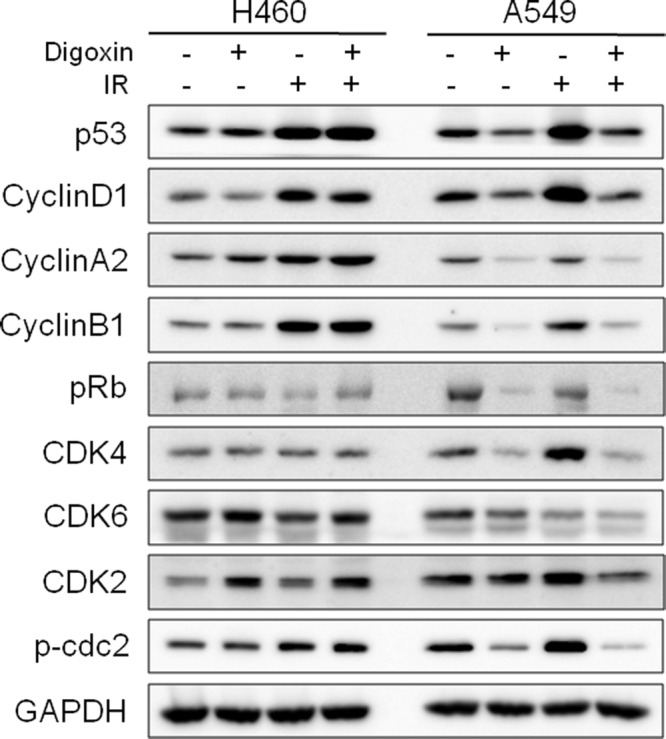
Digoxin affects the reduction in cell cycle related proteins in A549 cells Western blot analysis of protein expression levels for p53, cyclins (cyclin D1, cyclin A2, cyclin B1), pRb, and CDKs (CDK4, CDK6, CDK2, p-cdc2) following treatment of digoxin, IR, and a combination of both. H460 and A549 cells were pretreated with 40 nM digoxin for 24 h, and then irradiated with 6 Gy IR.

To elucidate the effect of digoxin on accumulation of IR-induced cell cycle proteins, we examined the expression of related proteins by Western blotting. Treatment with digoxin for 48 h resulted in reduced levels of p53, cyclins (cyclin D1, cyclin A2, and cyclin B1), CDKs (CDK4, CDK6, and p-cdc2), and pRb in A549 cells, but not in H460 cells (Figure 3). Moreover, the combination of digoxin with IR strongly reduced the levels of proteins up-regulated after IR (p53, cyclin D1, cyclin B1, CDK4, and p-cdc2) in A549 cells. However, digoxin did not affect the expression level of IR-induced proteins (p53, cyclin D1, cyclin A2, cyclin B1, and p-cdc2) in H460 cells. Thus, digoxin attenuates IR-induced expression of cell cycle checkpoint proteins in radioresistant A549 cells.

### Combined treatment with digoxin and IR results in sustained DNA damage via accumulation of p-ATM and reduction in repair proteins in A549 cells

**Figure 4:**
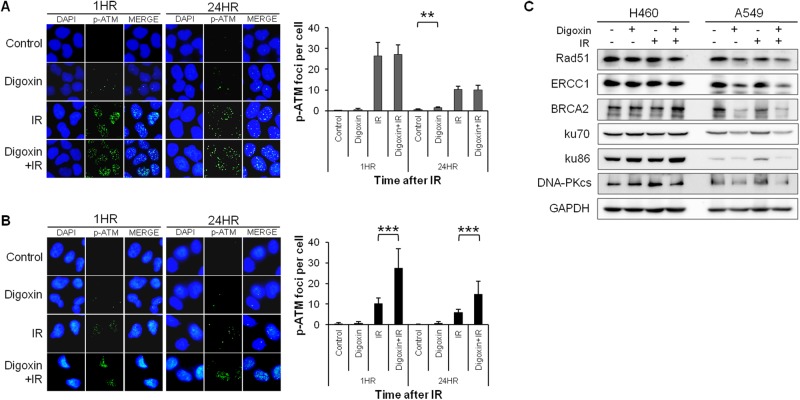
Digoxin prolongs IR-induced accumulation of p-ATM foci and reduces expression of repair proteins in radioresistant A549 cells compared with H460 cells The p-ATM foci formation in **(A)** H460 and** (B)** A549 cells treated with 40 nM digoxin, 6 Gy radiation, or both, and stained for p-ATM at 1 or 24 h after IR. Graph represents the quantitation of cells with positive p-ATM foci. Bars represent ± S.D.; **P<0.01, ***P<0.001. (**C**) Western blot analysis of expression levels of HR repair proteins (Rad51, ERCC1, and BRCA2) and NHEJ repair proteins (ku70, ku86, and DNA-PKcs) in both cell types.

We analyzed the effect of digoxin on DNA damage by assessing the presence of the DNA damage marker, p-ATM, by immunofluorescence staining. As shown in Figure 4A,B, digoxin alone did not affect the p-ATM foci formation in H460 and A549 cells. IR triggered the accumulation of p-ATM foci in both radioresistant A549 cells and radiosensitive H460 cells, although to a much higher level in the latter (*P*<0.001; compared with control). Combination of IR with digoxin treatment did not increase the foci formation compared with IR alone in H460 cells, but it enhanced p-ATM foci formation in A549 cells up to 24 h after IR (*P*<0.001; compared with IR alone). To investigate whether digoxin affected DNA repair pathways, we examined the expression levels of homologous recombination (HR) repair proteins (Rad51, ERCC1, and BRCA2) and non-homologous end joining (NHEJ) repair proteins (ku70, ku86, and DNA-PKcs) in H460 and A549 cells by Western blot analysis. In radiosensitive H460 cells, digoxin did not affect the level of repair proteins (Figure 4C). However, digoxin reduced expression of proteins involved in the HR repair pathway (Rad51, ERCC1, and BRCA2) and the NHEJ repair pathway (ku70, ku86, and DNA-PKcs) in radioresistant A549 cells. Hence, digoxin strongly potentiates the induction of DNA damage by IR treatment and decreases levels of repair proteins in radioresistant A549 cells, but not in radiosensitive H460 cells.

## Discussion

Digoxin, a well-known cardiac glycoside, is widely used in the treatment of cardiac failure, and it also inhibits the growth of STK11 mutant NSCLC cells [14]. As previously noted, digoxin reduced EGFR and STAT3 activity via suppression of Src activity [26] and down-regulated VEGF levels via the inhibition of HIF-1α under hypoxic conditions [27]. Although radiation-sensitive efficacy by digoxin has not yet been clarified, several lines of evidence have suggested that digoxin may increase radiosensitivity by reducing radioresistance-related proteins (e.g. Src, HIF-1α) [26,27]. This study demonstrated that a novel function of digoxin, the inhibition of PP2A, enhances radiosensitivity of radioresistant lung cancer cells *in vitro* and *in vivo*.

PP2A exists as a heterodimeric or heterotrimeric holoenzyme consisting of catalytic (C), scaffold (A), and regulatory (B) subunits that regulate the activity for either inhibitory or stimulatory effects on cell growth [28]. Although the dual activity of PP2A continues to be a controversial topic, inhibition of PP2A is of interest as a method to target tumors resistant to conventional treatments. Several studies have suggested that inhibition of PP2A induces anticancer effects through the activation of the JNK pathway [29], and the modulation of the PI3K/Akt pathway [30]. We found that digoxin increased the anticancer effect (Figure 1A) and decreased PP2A/A protein levels (Figure 2) in radioresistant A549 to a degree greater than radiosensitive H460 cells and xenografts. Thus, our data reveal that digoxin functions as a PP2A inhibitor to enhance anticancer effect in radioresistant A549 cells and xenografts. Interestingly, slowly growing tumors are particularly resistant to radiotherapy [31,32] and inhibition of PP2A could convert resistant tumor cells into a more sensitive phenotype by accelerating the cell cycle. Consistent with previous reports, the present study showed that PP2A inhibition by digoxin enhanced radiation response in slowly growing A549 cells and xenografts but not fast growing H460 cells (Figure 1). Next, the evidence from multiple lines of investigation in our study confirmed that digoxin radiosensitized A549 cells via PP2A inhibition. Compared with radiosensitive H460 cells, IR increased the expression of PP2A in radioresistant A549 cells, allowing more cells to stop at the cell cycle checkpoint (Figure 2). Digoxin attenuated IR-induced PP2A protein levels and led IR-induced damaged cells to progress through the cell cycle. Furthermore, digoxin reduced cell cycle checkpoint proteins (p53, cyclin D1, cyclin B1, CDK4, and p-cdc2), which may support cell cycle progression (Figure 3). Previous reports have suggested that PP2A inhibition increases DNA damage by enhancing γH2AX foci retention [12] and inhibition of HR repair of IR-induced DNA damage in U251 cells [13]. PP2A suppressed autophosphorylation of ATM [11] and a combination treatment with IR and PP2A inhibitor enhanced IR-induced DNA damage by autophosphorylation of ATM. In our study, digoxin significantly enhanced IR-induced p-ATM foci formation and prolonged the duration of p-ATM foci expression in radioresistant A549 cells (Figure 4B). In addition, digoxin suppressed the expression of repair proteins, reducing the recovery of DNA damage caused by the combination treatment (Figure 4C). Collectively, these results suggest that the radiosensitizing effect by digoxin may be related to cell cycle regulation and DNA damage repair pathways. However, the exact radiosensitive mechanism of digoxin via inhibition of PP2A protein in radioresistant A549 cell line remains to be further studied.

In conclusion, these findings represent the first evidence that digoxin acts as a PP2A inhibitor and enhances the radiosensitivity of radioresistant NSCLC cells. Our data support further evaluation of digoxin as well as preclinical exploration of the radiosensitizing properties of digoxin and other PP2A inhibitors.

## Abbreviations

ATM: Ataxia telangiectasia mutated
BRCA2: breast cancer 2
CDK: cyclin-dependent kinase
DNA-PKcs: DNA-dependent protein kinase
EGFR: epidermal growth factor receptor
ERCC1: excision repair cross-complementing 1
HIF-1α: hypoxia-inducible factor 1-alpha
HR: homologous recombination
IR: ionizing radiation
JNK: c-Jun N-terminal kinase
NF-κB: Nuclear factor kappa-light-chain-enhancer of activated B cells
NHEJ: non-homologous end joining
NSCLC: non-small-cell lung cancer
PP2A: protein phosphatase 2A
STAT3: signal transducer and activator of transcription 3
STK11: Serine/threonine kinase 11
VEGF: vascular endothelial growth factor
γH2AX: phosphorylated histone H2AX

## References

[1] Torre L.A., Sauer A.M., Chen M.S. Jr, Kagawa-Singer M., Jemal A. and Siegel R.L. (2016) Cancer statistics for Asian Americans, Native Hawaiians, and Pacific Islanders, 2016: converging incidence in males and females. CA Cancer J. Clin. 66, 182–202 26766789 10.3322/caac.21335 PMC5325676

[2] Chang J.Y., Kestin L.L., Barriger R.B., Chetty I.J., Ginsburg M.E., Kumar S. (2014) ACR Appropriateness Criteria® nonsurgical treatment for locally advanced non-small-cell lung cancer: good performance status/definitive intent. Oncology (Williston Park) 28, 706–710 25140629

[3] Chen G.Z., Zhu H.C., Dai W.S., Zeng X.N., Luo J.H. and Sun X.C. (2017) The mechanisms of radioresistance in esophageal squamous cell carcinoma and current strategies in radiosensitivity. J. Thorac. Dis. 9, 849–859 28449496 10.21037/jtd.2017.03.23 PMC5394057

[4] Sethi G., Shanmugam M.K., Ramachandran L., Kumar A.P. and Tergaonkar V. (2012) Multifaceted link between cancer and inflammation. Biosci. Rep. 32, 1–15 21981137 10.1042/BSR20100136

[5] Chai E.Z., Siveen K.S., Shanmugam M.K., Arfuso F. and Sethi G. (2015) Analysis of the intricate relationship between chronic inflammation and cancer. Biochem. J. 468, 1–15 25940732 10.1042/BJ20141337

[6] Seshacharyulu P., Pandey P., Datta K. and Batra S.K. (2013) Phosphatase: PP2A structural importance, regulation and its aberrant expression in cancer. Cancer Lett. 335, 9–18 23454242 10.1016/j.canlet.2013.02.036 PMC3665613

[7] Susila A., Chan H., Loh A.X., Phang H.Q., Wong E.T., Tergaonkar V. (2010) The POPX2 phosphatase regulates cancer cell motility and invasiveness. Cell Cycle 9, 179–187 20016286 10.4161/cc.9.1.10406

[8] Liu H., Gu Y., Wang H., Yin J., Zheng G., Zhang Z. (2015) Overexpression of PP2A inhibitor SET oncoprotein is associated with tumor progression and poor prognosis in human non-small cell lung cancer. Oncotarget 6, 14913–14925 25945834 10.18632/oncotarget.3818 PMC4558125

[9] Chew J., Biswas S., Shreeram S., Humaidi M., Wong E.T., Dhillion M.K. (2009) WIP1 phosphatase is a negative regulator of NF-kappaB signalling. Nat. Cell Biol. 11, 659–666 19377466 10.1038/ncb1873

[10] Mi J., Bolesta E., Brautigan D.L. and Larner J.M. (2009) PP2A regulates ionizing radiation-induced apoptosis through Ser46 phosphorylation of p53. Mol. Cancer Ther. 8, 135–140 19139122 10.1158/1535-7163.MCT-08-0457

[11] Goodarzi A.A., Jonnalagadda J.C., Douglas P., Young D., Ye R., Moorhead G.B. (2004) Autophosphorylation of ataxia-telangiectasia mutated is regulated by protein phosphatase 2A. EMBO J. 23, 4451–4461 15510216 10.1038/sj.emboj.7600455 PMC526470

[12] Chowdhury D., Keogh M.C., Ishii H., Peterson C.L., Buratowski S. and Lieberman J. (2005) gamma-H2AX dephosphorylation by protein phosphatase 2A facilitates DNA double-strand break repair. Mol. Cell 20, 801–809 16310392 10.1016/j.molcel.2005.10.003

[13] Gordon I.K., Lu J., Graves C.A., Huntoon K., Frerich J.M., Hanson R.H. (2015) Protein phosphatase 2A inhibition with LB100 enhances radiation-induced mitotic catastrophe and tumor growth delay in glioblastoma. Mol. Cancer Ther. 14, 1540–1547 25939762 10.1158/1535-7163.MCT-14-0614 PMC4497833

[14] Kim N., Yim H.Y., He N., Lee C.J., Kim J.H., Choi J.S. (2016) Cardiac glycosides display selective efficacy for STK11 mutant lung cancer. Sci. Rep. 6, 29721 27431571 10.1038/srep29721 PMC4949473

[15] Marcus F.I., Kapadia G.J. and Kapadia G.G. (1964) The metabolism of digoxin in normal subjects. J. Pharmacol. Exp. Ther. 145, 203–209 14214418

[16] Iisalo E. (1977) Clinical pharmacokinetics of digoxin. Clin. Pharmacokinet. 2, 1–16 322907 10.2165/00003088-197702010-00001

[17] Newman R.A., Yang P., Pawlus A.D. and Block K.I. (2008) Cardiac glycosides as novel cancer therapeutic agents. Mol. Interv. 8, 36–49 18332483 10.1124/mi.8.1.8

[18] Durlacher C.T., Chow K., Chen X.W., He Z.X., Zhang X., Yang T. (2015) Targeting Na(+)/K(+) -translocating adenosine triphosphatase in cancer treatment. Clin. Exp. Pharmacol. Physiol. 42, 427–443 25739707 10.1111/1440-1681.12385

[19] Stenkvist B. (1999) Is digitalis a therapy for breast carcinoma? Oncol. Rep. 6, 493–496 10203580

[20] Trenti A., Grumati P., Cusinato F., Orso G., Bonaldo P. and Trevisi L. (2014) Cardiac glycoside ouabain induces autophagic cell death in non-small cell lung cancer cells via a JNK-dependent decrease of Bcl-2. Biochem. Pharmacol. 89, 197–209 24630927 10.1016/j.bcp.2014.02.021

[21] Liu M., Feng L.X., Sun P., Liu W., Wu W.Y., Jiang B.H. (2016) A novel bufalin derivative exhibited stronger apoptosis-inducing effect than bufalin in A549 lung cancer cells and lower acute toxicity in mice. PLoS ONE 11, e0159789 27459387 10.1371/journal.pone.0159789 PMC4961401

[22] Elbaz H.A., Stueckle T.A., Wang H.Y., O’Doherty G.A., Lowry D.T., Sargent L.M. (2012) Digitoxin and a synthetic monosaccharide analog inhibit cell viability in lung cancer cells. Toxicol. Appl. Pharmacol. 258, 51–60 22037315 10.1016/j.taap.2011.10.007 PMC3254807

[23] Nasu S., Milas L., Kawabe S., Raju U. and Newman R. (2002) Enhancement of radiotherapy by oleandrin is a caspase-3 dependent process. Cancer Lett. 185, 145–151 12169388 10.1016/s0304-3835(02)00263-x

[24] Wang L., Raju U., Milas L., Molkentine D., Zhang Z., Yang P. (2011) Huachansu, containing cardiac glycosides, enhances radiosensitivity of human lung cancer cells. Anticancer Res. 31, 2141–2148 21737634

[25] Verheye-Dua F. and Bohm L. (1998) Na+, K+-ATPase inhibitor, ouabain accentuates irradiation damage in human tumour cell lines. Radiat. Oncol. Invest. 6, 109–119 10.1002/(SICI)1520-6823(1998)6:3<109::AID-ROI1>3.0.CO;2-1 9652909

[26] Lin S.Y., Chang H.H., Lai Y.H., Lin C.H., Chen M.H., Chang G.C. (2015) Digoxin suppresses tumor malignancy through inhibiting multiple Src-related signaling pathways in non-small cell lung cancer. PLoS ONE 10, e0123305 25955608 10.1371/journal.pone.0123305 PMC4425490

[27] Zhang H., Qian D.Z., Tan Y.S., Lee K., Gao P., Ren Y.R. (2008) Digoxin and other cardiac glycosides inhibit HIF-1alpha synthesis and block tumor growth. Proc. Natl. Acad. Sci. U.S.A. 105, 19579–19586 19020076 10.1073/pnas.0809763105 PMC2604945

[28] Schonthal A.H. (2001) Role of serine/threonine protein phosphatase 2A in cancer. Cancer Lett. 170, 1–13 11448528 10.1016/s0304-3835(01)00561-4

[29] Li W., Chen Z., Gong F.R., Zong Y., Chen K., Li D.M. (2011) Growth of the pancreatic cancer cell line PANC-1 is inhibited by protein phosphatase 2A inhibitors through overactivation of the c-Jun N-terminal kinase pathway. Eur. J. Cancer 47, 2654–2664 21958460 10.1016/j.ejca.2011.08.014

[30] Hong C.S., Ho W., Zhang C., Yang C., Elder J.B. and Zhuang Z. (2015) LB100, a small molecule inhibitor of PP2A with potent chemo- and radio-sensitizing potential. Cancer Biol. Ther. 16, 821–833 25897893 10.1080/15384047.2015.1040961 PMC4623051

[31] Lee J.Y., Kim M.S., Kim E.H., Chung N. and Jeong Y.K. (2016) Retrospective growth kinetics and radiosensitivity analysis of various human xenograft models. Lab. Anim. Res. 32, 187–193 28053611 10.5625/lar.2016.32.4.187 PMC5206224

[32] Moore N. and Lyle S. (2011) Quiescent, slow-cycling stem cell populations in cancer: a review of the evidence and discussion of significance. J. Oncol. 2011, 10.1155/2011/396076 PMC2948913 20936110

